# Supplementary material for: Atg2 Regulates Cellular and Humoral Immunity in Drosophila
Source: Insects. 2023 Aug 14;14(8):706. doi: 10.3390/insects14080706 (PMC10455222; doi:10.3390/insects14080706)
Supplement: Supplementary file 1 [file insects-14-00706-s001.zip › insects-2506658-supplementary.pdf]

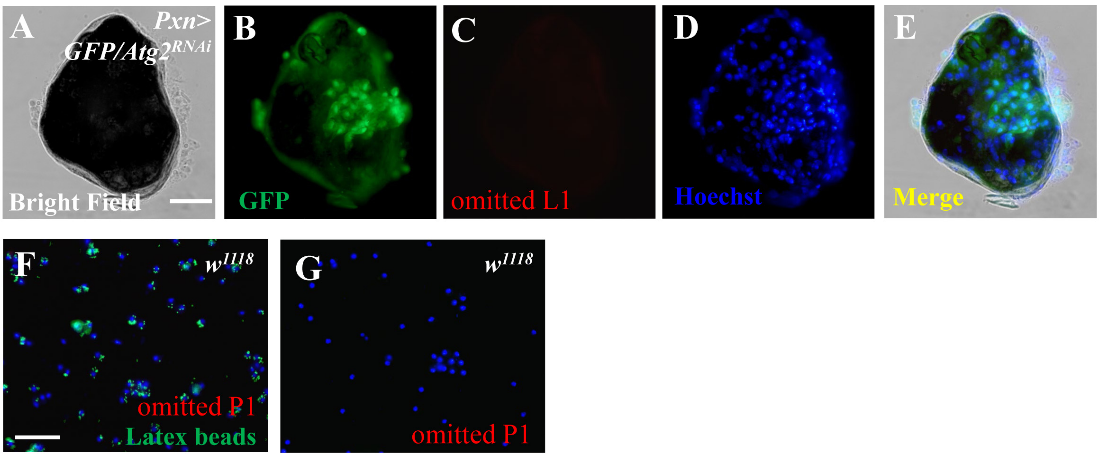

Figure S1. The negative controls for L1 staining and P1 staining. (**A-E**) Melanotic nodules from *Pxn > GFP/Atg2*^{RNAi} larvae were stained with omitted anti-L1 antibodies and Alexa Fluor 568-conjugated secondary antibodies. (**F-G**) Hemocytes from w^{1118} larvae with or without being injected with latex beads (green) were stained with omitted anti-P1 antibodies and Alexa Fluor 568-conjugated secondary antibodies. However, no red fluorescence was observed in C,F,G, indicating that the red fluorescence signals from L1 or P1 staining were specific. scale bar: 50 μm.

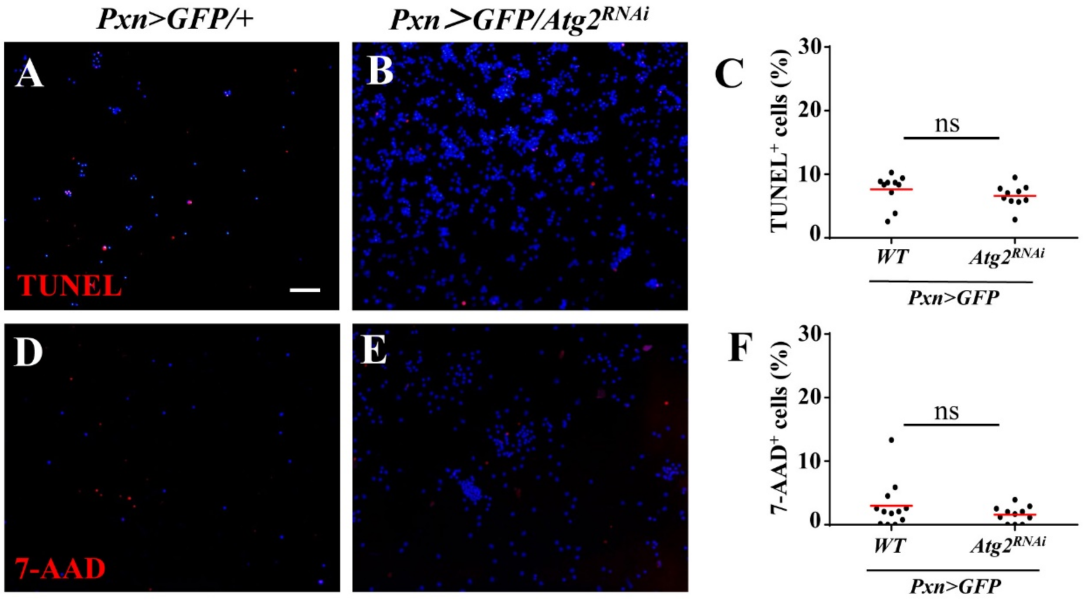

Figure S2. Inhibiting *Atg2*did not induce cell apoptosis or death. (**A-E**) Cell apoptosis (**A-B**) and cell death (**D-E**) were examined with TUNEL and 7-AAD, respectively, in *Pxn* > *GFP*/+ and *Pxn* > *GFP*/*Atg2^{RNAi}* larvae. The quantifications (**C,F**) showed that there was no significant difference between the control and experimental groups. ns, not significant; scale bar: 50 μm.

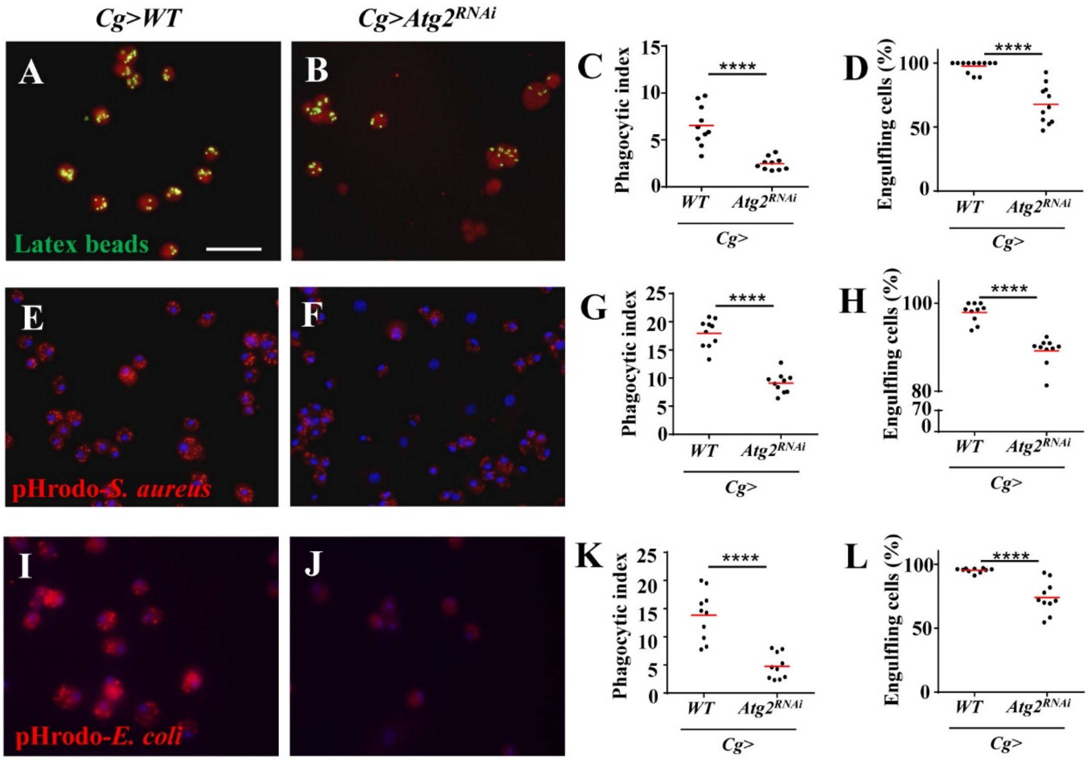

Figure S3. Phagocytosis was disrupted upon inhibition of *Atg2*. (**A-L**) Latex beads (**A-B**), pHrodo-*S. aureus* (**E-F**) and pHrodo-*E. coli* (**I-J**) were injected into *Cg* > *WT* and *Cg* > *Atg2*^{RNAi} larvae, respectively. The quantification analysis showed that the average phagocytosed particles (**C,G,K**) and the percentage of engulfing hemocytes (**D,H,L**) significantly decreased when *Atg2* was knocked down in hemocytes and fat bodies. ****p<0.0001; scale bar: 25 μm.
